# 

**Published:** 1905-08

**Authors:** 


### ATLANTA JOURNAL-RECORD OF MEDICINE.

AUG. 15. 1905

Successor to Atlanta Medical and Surgical Journal, Established 1855, and Southern Medical Record, Established 1870.

Vol. VII. Atlanta, Ga., August, 1905. No. 5.

